# Tumour-associated and non-tumour-associated bacteria co-abundance groups in colorectal cancer

**DOI:** 10.1186/s12866-024-03402-5

**Published:** 2024-07-03

**Authors:** Yuxuan Liang, Qingrong Zhang, Jing Yu, Wenyan Hu, Sihua Xu, Yiyuan Xiao, Hui Ding, Jiaming Zhou, Haitao Chen

**Affiliations:** 1 School of Public Health (Shenzhen), Sun Yat-sen University, Guangzhou, China; 2 School of Public Health (Shenzhen), Shenzhen Campus of Sun Yat-sen University, Shenzhen, China; 3 Department of General Surgery, The Sixth Affiliated Hospital, Sun Yat-sen University, Guangzhou, Guangdong China; 4 Guangdong Provincial Key Laboratory of Colorectal and Pelvic Floor Diseases, The Sixth Affiliated Hospital, Sun Yat-sen University, Guangzhou, Guangdong China; 5 Department of General Surgery, First Affiliated Hospital of Jinan University, Guangzhou, China

**Keywords:** Colorectal Cancer, 16S rRNA sequencing, Mucosal tissue, Microbiota classification, Biomarkers

## Abstract

**Background & aims:**

Gut microbiota is closely related to the occurrence and development of colorectal cancer (CRC). However, the differences in bacterial co-abundance groups (CAGs) between tumor tissue (TT) and normal tissue (NT), as well as their associations with clinical features, are needed to be clarified.

**Methods:**

Bacterial 16 S rRNA sequencing was performed by using TT samples and NT samples of 251 patients with colorectal cancer. Microbial diversity, taxonomic characteristics, microbial composition, and functional pathways were compared between TT and NT. Hierarchical clustering was used to construct CAGs.

**Results:**

Four CAGs were grouped in the hierarchical cluster analysis. CAG 2, which was mainly comprised of pathogenic bacteria, was significantly enriched in TT samples (2.27% in TT vs. 0.78% in NT, *p* < 0.0001). CAG 4, which was mainly comprised of non-pathogenic bacteria, was significantly enriched in NT samples (0.62% in TT vs. 0.79% in NT, *p* = 0.0004). In addition, CAG 2 was also significantly associated with tumor microsatellite instability (13.2% in unstable vs. 2.0% in stable, *p* = 0.016), and CAG 4 was positively correlated with the level of CA199 (*r* = 0.17, *p* = 0.009).

**Conclusions:**

Our research will deepen our understanding of the interactions among multiple bacteria and offer insights into the potential mechanism of NT to TT transition.

**Supplementary Information:**

The online version contains supplementary material available at 10.1186/s12866-024-03402-5.

## Introduction

The global incidence of colorectal cancer (CRC) has increased rapidly and in China, it ranks second among all malignant tumors [1–3]. Traditional risk factors for CRC include family history, inflammatory bowel disease, processed meat intake, diabetes, obesity, smoking, and alcohol consumption [4, 5].

Previous studies found that changes in gut microbiota such as *Streptococcus bovis*, *Helicobacter pylori*, *Bacteroides fragilis*, *Enterococcus faecalis*, *Clostridium septicum*, *Fusobacterium spp*, and *Escherichia coli*, were closely related to the occurrence of gastrointestinal cancer [6]. However, for several microorganisms such as *Fusobacterium* species, the association of their abundance with human colon cancer was not consistent in all reports and lacked a clear conclusion [7, 8]. Abreu et al’s research indicated that the inconsistency between studies may be due to the heterogeneity of microbial or host response levels [9]. Therefore, Flemer et al. proposed that combinations or co-abundance groups (CAGs) of organisms may be more operative to express the relationship between microbiota and disease, rather than to represent a one organism-one disease model [10]. In Flemer’s study, they found that it was feasible to use a combination of several bacteria (or microbiome characteristics) in the stool microbiota of CRC patients as a marker to detect the disease [10]. The utility of CAGs was further confirmed in investigating the association between the microbiota community of tongue coating and the prognosis of gastric cancer [11]. Recently, a study conducted in Australia demonstrated that the construction of an oncomicrobial community subtype, similar to the CAGs in Flemer’s paper, using tumor tissue (TT) and normal tissue (NT) samples can effectively predict the prognosis of CRC [12]. However, the performance of CAGs in Chinese CRC patients is not clear.

In this study, we conducted a cross-sectional study of the colon microbiome in 492 mucosal samples (245 TT and 247 TT) from 248 patients undergoing CRC surgery. We found that the diversity of microbiota between TT and NT was significantly different, with each group exhibiting distinct taxonomic profiling and discriminant taxa. In addition, the intratumor microbiota of CRC could be categorized into four CAGs and CRC patients could be further divided into 6 distinct groups based on four CAGs. This study may provide novel insights into the dynamics of bacterial communities during the conversion of NT to TT.

## Materials and methods

### Study participants

Samples were obtained from patients undergoing surgical treatment for colorectal cancer. Ultimately, a total of 251 patients diagnosed with colorectal cancer were recruited at the Sixth Affiliated Hospital of Sun Yat-sen University from 2015 to 2021. Detailed information on these samples is provided in Table 1.

**Table 1:** The demographic characteristics of all the enrolled samples

Characteristics	Patients (248)
**Gender**	
Female	97(39.11%)
Male	151(60.89%)
**Site**	
Left hemicolon	84(33.87%)
Rectum	109(43.95%)
Right hemicolon	55(22.18%)
**Stage**	
Advanced	118(47.58%)
Early	130(52.42%)
**Gross**	
Infiltration	2(0.81%)
Mass	67(27.02%)
Ulcer	177(71.37%)
NA	2(0.81%)
**Differentiation**	
High	38(15.32%)
Low	179(72.18%)
Median	17(6.85%)
NA	14(5.65%)
**Ki67**	
50% or more	127(51.21%)
Less than 50%	121(48.79%)
**Microsatellite**	
Unstable	17(6.85%)
Stable	230(92.74%)
NA	1(0.4%)
**Age** (years)	62.94 ± 12.29
**Height** (cm)	162.89 ± 7.79
**Weight** (kg)	60.16 ± 10.39
**BMI** (kg/m²)	22.63 ± 3.27
**CEA** (ng/ml)	87.63 ± 1020.43
**CA199** (U/ml)	74.61 ± 451
**CA125** (U/ml)	17.97 ± 32.06
**CA153** (U/ml)	10.39 ± 5.83
**AFP** (ng/ml)	3.37 ± 8.9

Note: Data are shown as means ± SD

Tumor specimens were obtained using a sterile scalpel blade within 1 h following surgical resection, and the normal tissue samples were obtained at a standardized distance of over three centimeters from the tumor margin. All samples were promptly frozen in liquid nitrogen and stored at -80 °C. Inclusion criteria encompassed patients aged 18 years or older without contraindications to colorectal cancer resection. The exclusion criteria include the use of antibiotics or probiotics within one month, radiotherapy, chemotherapy, intestinal obstruction, and concurrent other severe organic diseases. The stage of CRC was classified according to the 8th edition of the American Joint Committee on Cancer (AJCC) TNM staging system. The protocol of human sample usage and the informed consent was approved by the Ethical Review Board of the Sixth Affiliated Hospital of Sun Yat-sen University (2020ZSLYEC-101).

### DNA extraction and PCR amplification

Total microbial genomic DNA was extracted from TT and NT samples using FastDNA Spin Kit for Soil (MP Biomedicals) according to the manufacturer’s instructions. The quality and concentration of DNA were determined by 1.0% agarose gel electrophoresis and a NanoDrop^®^ ND-2000 spectrophotometer (Thermo Scientific Inc., USA) and kept at -80℃ prior to further use. The hypervariable region *V3-V4* of the bacterial 16 S rRNA gene was amplified with primer pairs *338 F (5’-ACTCCTACGGGAGGCAGCAG-3’) and 806R(5’-GGACTACHVGGGTWTCTAAT-3’)* [13] by an ABI GeneAmp^®^ 9700 PCR thermocycler (ABI, CA, USA). The PCR reaction mixture including 4 µL 5 × Fast Pfu buffer, 2 µL 2.5 mM dNTPs, 0.8 µL each primer (5 µM), 0.4 µL Fast Pfu polymerase, 10 ng of template DNA, and ddH_2_O to a final volume of 20 µL. PCR amplification cycling conditions were as follows: initial denaturation at 95 ℃ for 3 min, followed by 27 cycles of denaturing at 95 ℃ for 30 s, annealing at 55 ℃ for 30 s, and extension at 72 ℃for 45 s, and single extension at 72 ℃ for 10 min, and end at 4 ℃. All samples were amplified in triplicate. The PCR product was extracted from 2% agarose gel and purified using the AxyPrep DNA Gel Extraction Kit (Axygen Biosciences, Union City, CA, USA) according to manufacturer’s instructions and quantified using Quantus™ Fluorometer (Promega, USA).

### Illumina MiSeq sequencing

Purified amplicons were pooled in equimolar amounts and paired-end sequenced on an Illumina MiSeq PE300 platform (Illumina, San Diego, USA) according to the standard protocols by Majorbio Bio-Pharm Technology Co. Ltd. (Shanghai, China).

### Statistical analysis

The raw data were processed using QIIME2 (Version 2021.8.0) to remove reads with insufficient repetitions (reads with less than 48 entries or fewer than 25 samples containing the reads) or readings shorter than 148 bp. Subsequently, the filtered reads were clustered into operational taxonomic units (OTUs) at a similarity threshold of 97%. To mitigate the potential impact of sequencing depth on subsequent alpha and beta diversity analyses, the sequence count in all samples was standardized to 16,864 sequences. As sequencing depth increased, the observed feature curves for both sample groups reached a plateau, indicating sufficient sequencing coverage. Taxonomic classification of each OTU was performed by comparing the sequences against SILVA database (version 138.1). Alpha diversity was assessed by calculating the ACE, Chao1, Observe, Pielou, Shannon, and Simpson indices. To compare the diversity differences among groups, Beta diversity was examined through principal coordinates analysis (PCoA) based on Bray-Curtis distance. Permutational multivariate analysis of variance using distance matrices (pMANOVA) was employed to assess the significance of beta diversity. Linear discriminant analysis Effect Size [14] (LEfSe) was used (http://galaxy.biobakery.org/) to identify key microorganisms associated with different groups, with an LDA threshold of 3.5. Bray-Curtis distance-based hierarchical clustering with Ward linkage method was utilized to construct CAGs, where only genera exhibiting a relative abundance exceeding 0.1% in TT and NT were used. For continuous variables, Mann-Whitney U test was employed to compare differences between groups, while Spearman rank correlation analysis was used for assessing correlations [15]. Prediction of Kyoto Encyclopedia of Genes and Genomes (KEGG) pathways was performed using Phylogenetic Investigation of Communities by Reconstruction of Unobserved States (PICRUSt) analysis. Statistical analyses and figures were conducted using R version 4.2.3 (R Foundation for Statistical Computing, Vienna, Austria.). A statistically significant difference was considered when the P value < 0.05.

## Results

### Baseline characteristics of participants

A total of 248 patients diagnosed with colorectal cancer were included in this study. Consequently, 245 TT samples and 247 NT samples from these enrolled patients were obtained for analysis. The demographic characteristics of all 248 enrolled patients are presented in Table 1.

### Comparison of the Microbial diversity between TT and NT

**Fig. 1:**
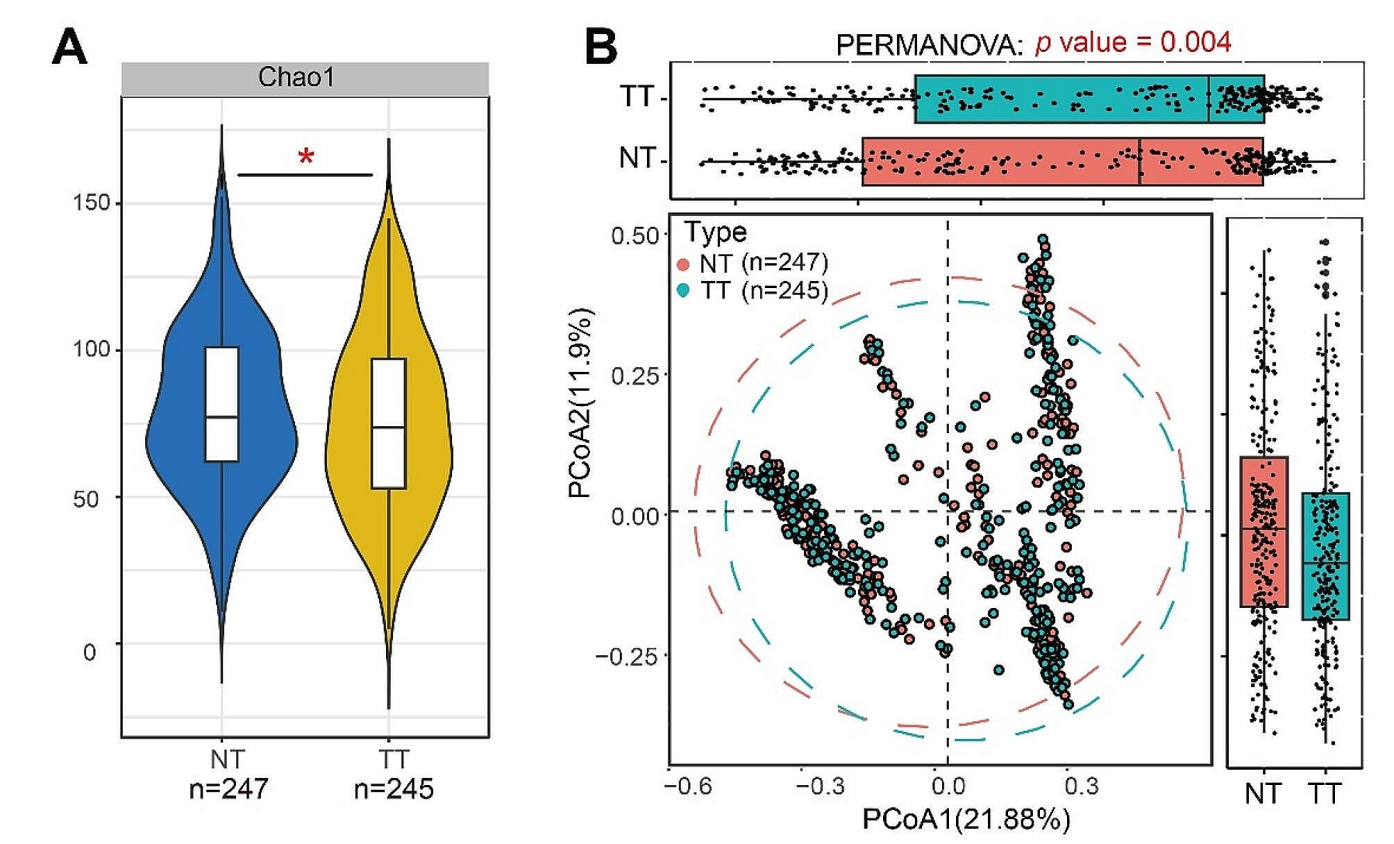
The microbial Alpha diversity and Beta diversity analysis in TT (Tumor Tissue) and NT (Normal Tissue). (**A**) Violin plots of Alpha diversity based on Chao1. (**B**) Beta diversity was calculated using Bray-Curtis by PCoA. The test method is Permanova *p < 0.05

In terms of alpha diversity, we employed ACE, Chao1, Observe, Pielou, Shannon, and Simpson indices to assess the species’ richness, evenness, and diversity of TT and NT. Based on the estimated results of the Chao1 index analysis (Fig. 1A; *p* = 0.048), we found that the alpha diversity of microbiota in NT was significantly higher than that of TT. Beta diversity was also significantly different between TT and NT (Fig. 1B; Permanova: Bray-Curtis *p* = 0.004).

### Taxonomic profiling and discriminant taxa between TT and NT

**Fig. 2:**
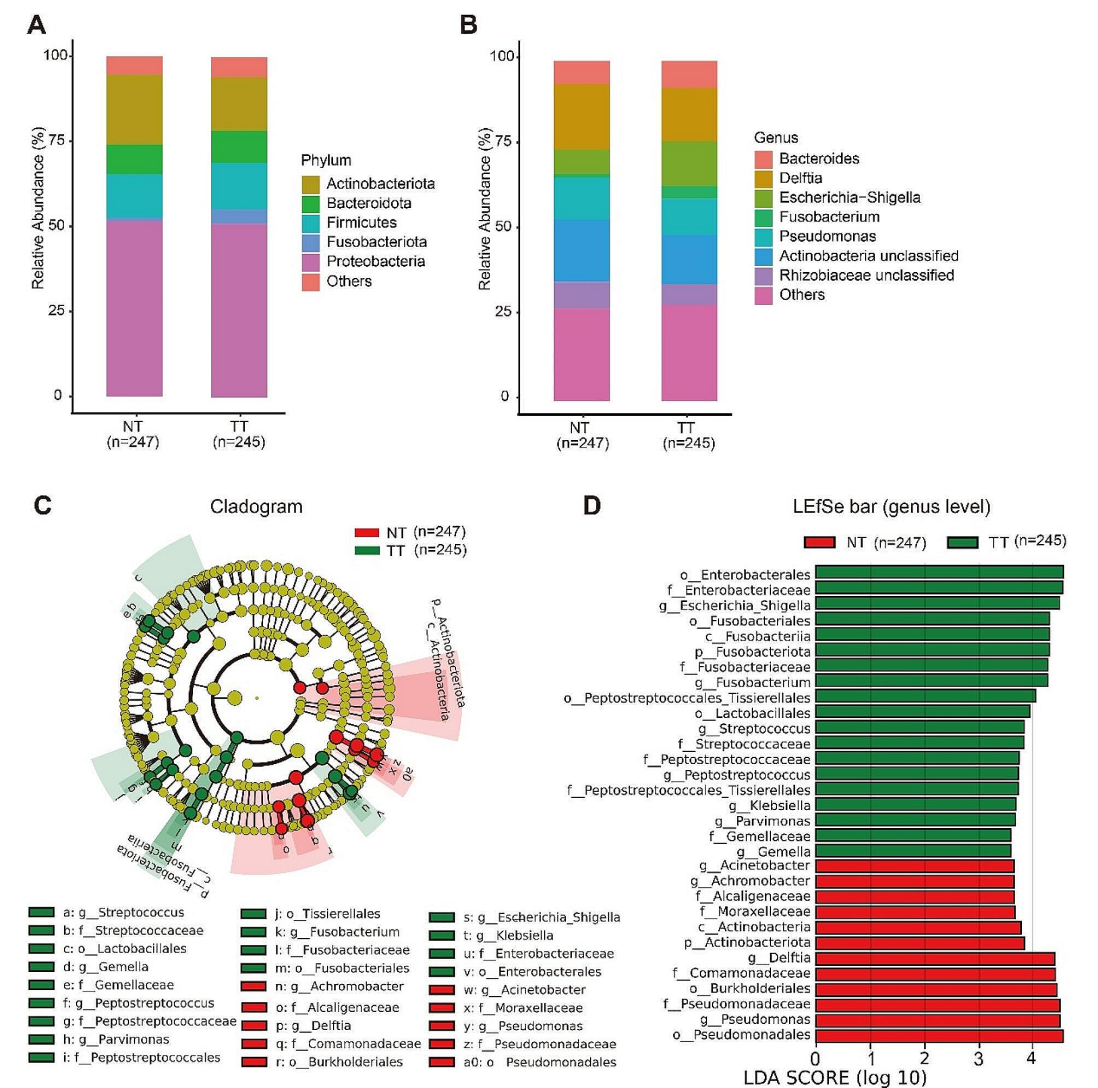
Characteristics of the microbiota in TT (Tumor Tissue) and NT (Normal Tissue). Each group Barplots of the relative abundance of the main bacterial taxa at (**A**) phylum, (**B**) genus level for the TT and NT. Cladogram (**C**) and Linear discriminant analysis effect size (LEfSe) analysis (**D**) showed the most abundant taxa from the phylum to the genus level among the TT and NT, LDA score threshold > 3.5

As illustrated in Fig. 2A and B, *Proteobacteria*, *Actinobacteriota*, *Firmicutes*, *Bacteroidota* and *Fusobacteriota* were five predominant phyla (Fig. 2A) while *Delftia*, *Actinobacteria unclassified*, *Pseudomonas*, *Bacteroidota*, *Escherichia-shigella*, *and Hizobiaceae unclassified* were six dominant genera (Fig. 2B).

LEfSe analyses were employed to identify significant microbial biomarkers across all taxa, with an LDA score threshold of > 3.5. As depicted in Fig. 2C and D, at the genus level, *Escherichia-Shigella*, *Fusobacterium*, *Streptococcus*, *Peptostreptococcus*, *Parvimonas, Klebsiella*, and *Gemella* were enriched in TT. While *Acinetobacter*, *Achromobacter*, *Delftia*, and *Pseudomonas* were enriched in NT. These findings suggest distinct microbiota compositions between TT and NT. In addition, we also identified the significant genera of different TMN stages through this LEfSe analyses (Supplementary Fig. 1A-B). In order to preserve the efficacy of each paired sample, we also performed differential abundance analysis on paired tumor tissues and paired normal tissues in terms of bacterial genus (Supplementary Table 1).

### Distribution of CAGs in TT and NT

**Fig. 3:**
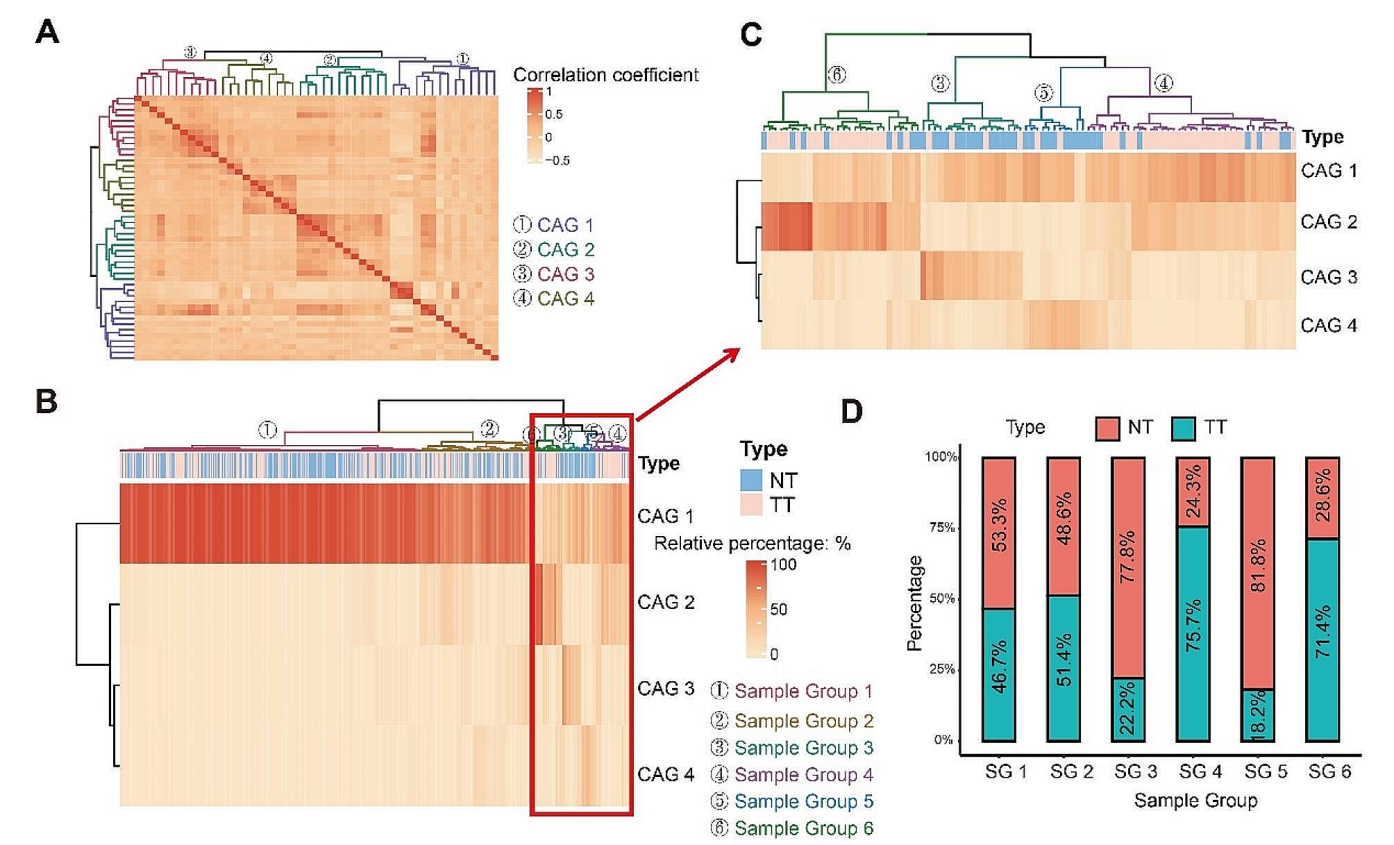
Cluster analysis. (**A**) Hierarchical Ward-linkage clustering based on the Spearman rank correlation coefficients of the genera with relative abundances greater than 0.1% in TT (Tumor Tissue) and NT (Normal Tissue). CAGs were defined based on the clusters in the tree. (**B**) Hierarchical Ward-linkage clustering based on the relative abundances of bacterial groups in TT and NT. Sample groups were defined based on the CAGs. (**C**) Part of B. (**D**) Barplots of distribution of TT and NT in sample groups

We performed hierarchical cluster analysis based on 47 genera with relative abundances exceeding 0.1%, resulting in the identification of four co-abundance groups (CAGs). The correlation of these 47 genera with four CAGs is shown in Fig. 3A and the detailed co-enriched genera of each CAG are shown in Table 2. By conducting sample-level cluster analysis using the abundance profiles of four CAGs, we identified six distinct sample groups (Fig. 3B). Notably, due to the high abundance of CAG 1 in sample group 1 and sample group 2, it was challenging to accurately discriminate TT from NT in these two sample groups. However, in the remaining four sample groups (sample group 3, sample group 4, sample group 5, and sample group 6), clear discrimination between TT and NT was observed (Fig. 3C). Sample group 4 and sample group 6 were primarily composed of TT, while sample group 3 and sample group 5 were predominantly composed of NT (Fig. 3D).

**Table 2:** The detailed co-enriched genera of each CAG

CAG 1	CAG 2	CAG 3	CAG 4
Achromobacter	Anaerococcus	Akkermansia	Acinetobacter
Bacteroides	Campylobacter	Alistipes	Aquabacterium
Chloroplast	Eikenella	Bifidobacterium	Bacillus
Delftia	Erysipelatoclostridium	Blautia	Chryseobacterium
Enterococcus	Fusobacterium	Collinsella	Lactobacillus
Escherichia-Shigella	Gemella	Dialister	Ralstonia
Klebsiella	Granulicatella	Faecalibacterium	Caulobacteraceae_unclassified
Proteus	Leptotrichia	Holdemanella	Clostridiaceae_unclassified
Pseudomonas	Parvimonas	Parabacteroides	Peptostreptococcaceae_unclassified
Actinobacteria_unclassified	Peptostreptococcus	Prevotella	Sphingomonadaceae_unclassified
Enterobacteriaceae_unclassified	Porphyromonas	Subdoligranulum	-
Lachnospiraceae_unclassified	Streptococcus	-	-
Rhizobiaceae_unclassified	-	-	-
Unknown	-	-	-

**Fig. 4:**
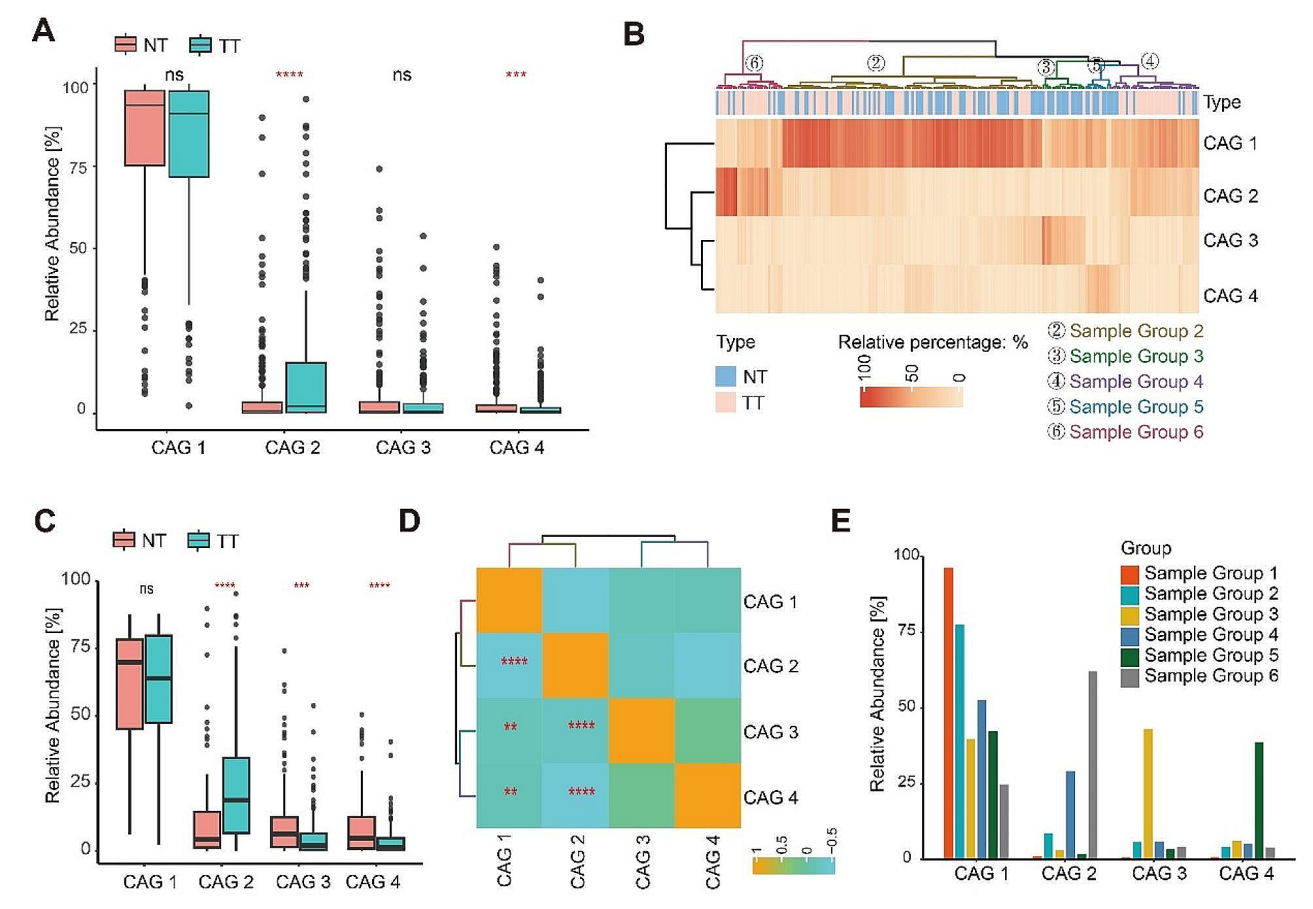
Characteristics of the CAGs and sample groups. (**A**) Boxplots of relative abundances of the four CAGs. (**B**) Hierarchical Ward-linkage clustering based on the relative abundances of CAGs in TT (Tumor Tissue) and NT (Normal Tissue). (**C**) Boxplots of relative abundances of the four CAGs. (**D**) Correlation coefficient matrix of CAGs based on Spearman rank correlation. (**E**) Barplots of distribution of CAGs in each sample group SG: Sample Group. ****p < 0.0001, ***p < 0.001, **p < 0.01, *p < 0.05

The relative abundance of four CAGs in TT and NT were illustrated in Fig. 4A. We found that CAG 2 exhibited significantly higher abundance in TT (median: 2.27% in TT vs. 0.78% in NT, *p* < 0.0001) while CAG 4 exhibited significantly higher abundance in NT (median: 0.62% in TT vs. 0.79% in NT, *p* = 0.0004). However, no significant differences were observed for CAG 1 and CAG 3. Interestingly, after removing sample group 1 which exhibited a predominant enrichment in CAG 1, CAG 3 displayed an increased abundance in NT (median: 1.9% in TT vs. 6.2% in NT, *p* = 0.0006, Fig. 4B and C) and exhibited an inverse correlation with CAG 2 (Fig. 4D). As shown in Fig. 4E, CAG 2 was highly abundant in sample group 4 and sample group 6 which mainly consisted of TT, while CAG 3 and CAG 4 were highly abundant in sample group 3 and sample group 5 which mainly consisted of NT.

### Association of CAGs with clinical features

**Fig. 5:**
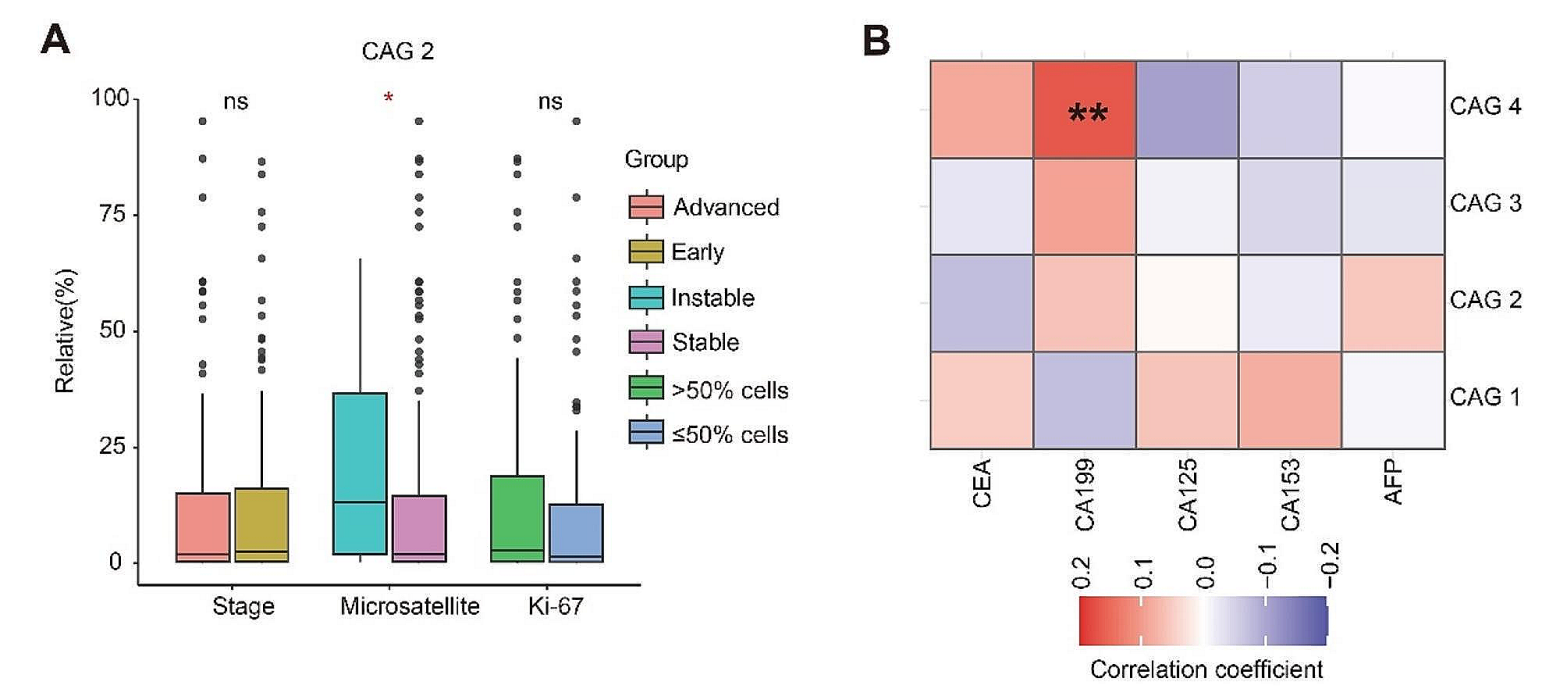
Association of CAGs with clinical features. (**A**) Boxplots of correlation between CAG 2 and tumor stage, microsatellite status, Ki-67. (**B**) Spearman rank correlation coefficient matrix heatmap between CAGs and CEA, CA199, CA125, CA153, AFP. ****p < 0.0001, ***p < 0.001, **p < 0.01, *p < 0.05

We further investigated the association of identified CAGs with clinical features in TT. Notably, we observed a significant association between CAG 2 and tumor microsatellite status. Specifically, a higher abundance of CAG 2 was found in samples with microsatellite instability (median: 13.2% in unstable vs. 2.0% in stable, *p* = 0.016, Fig. 5A). Furthermore, we evaluated the association of CAGs with tumor markers and specifically observed a positive correlation between CAG 4 and CA199 (*r* = 0.17, *p* = 0.009) (Fig. 5B).

### Functional analysis of microbiota in each group

**Fig. 6:**
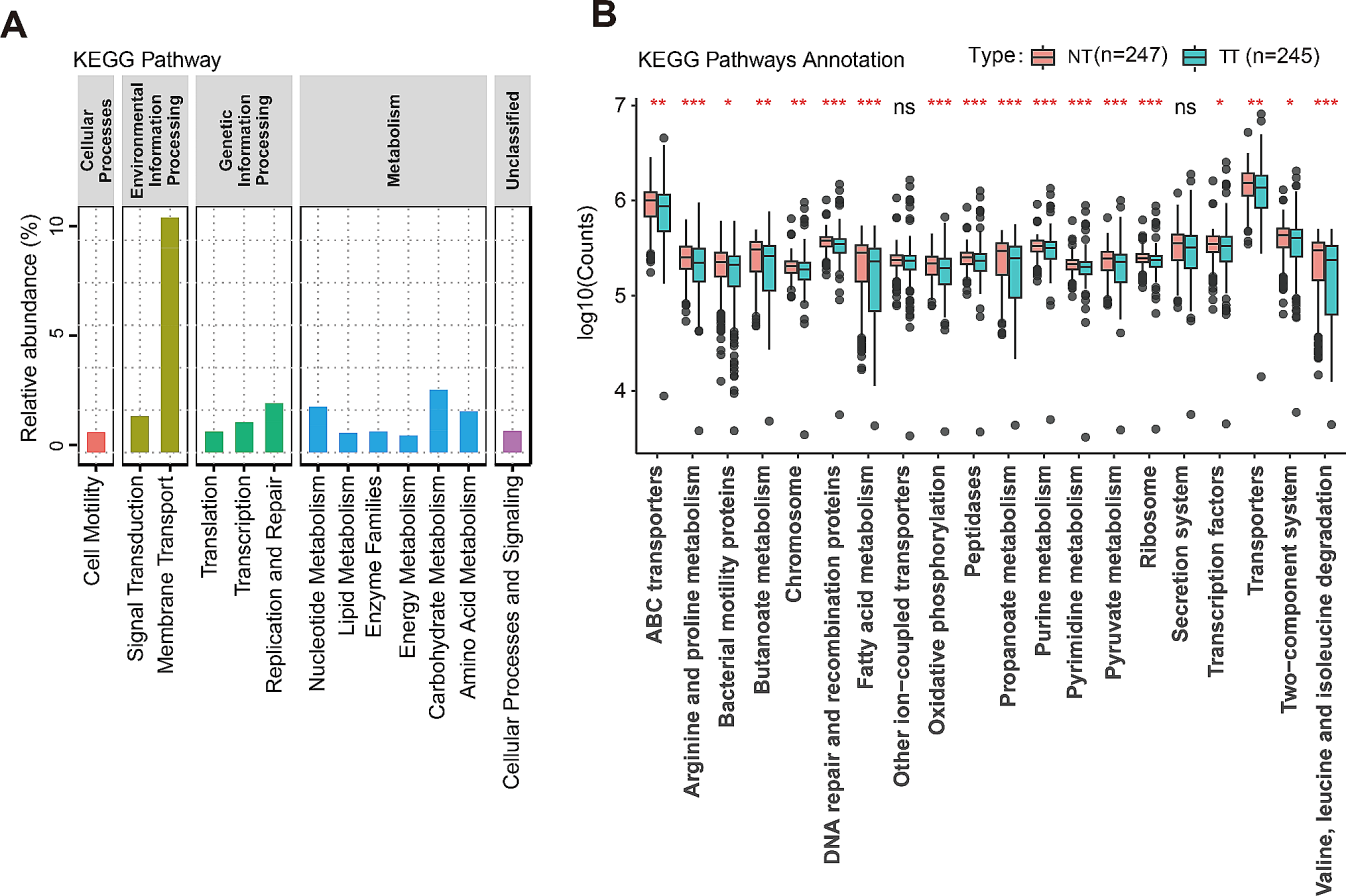
The function prediction of the two groups. (**A**) Barplots of the Kyoto Encyclopedia of Genes and Genomes (KEGG) pathways with an average relative abundance above 1% in TT and NT. (**B**) Boxplots of Differential Kyoto Encyclopedia of Genes and Genomes (KEGG) pathways were analyzed using PICRUSt for the TT and NT. The y-axis represents the counts of annotations to the pathways, using a base 10 logarithmic scale TT: Tumor Tissue, NT: Normal Tissue, ****p < 0.0001, ***p < 0.001, **p < 0.01, *p < 0.05

Finally, PICRUSt was employed to predict the KEGG pathways implicated in TT and NT. The KEGG pathways with an average relative abundance above 1% in all samples are shown in Fig. 6A. Notably, the membrane transport pathway constituted a substantial proportion, accounting for 13.8%. Of the 20 pathways with an average relative abundance above 1%, 18 of them were significantly upregulated in NT (Fig. 6B).

## Discussion

Tons of studies indicated that CRC-related microbiota can provide valuable insights into cancer occurrence, progression, and response to treatment [16]. Disparities between TT and NT primarily arise from individual taxonomic variations on the taxonomic profiling. In this study, by constructing community structures known as co-abundant groups (CAGs), we grouped all samples and examined the association of clinical characteristics with CAGs.

Compared with NT, a higher diversity of organisms in TT was observed as indicated by the Chao1 index of alpha diversity. PCoA analysis based on Bray-Curtis distance also demonstrated a significant differentiation between TT and NT. Similar findings were found in Loke’s studies (Loke et al., 2018) [17]. However, some studies have also reported non-significant differences in microbial diversity (α- and β-diversity) between TT and NT (Liu et al., 2021; Li M. et al., 2020) [18, 19]. These discrepancies can be partially attributed to variations in geographical location and tumor heterogeneity. Regarding taxonomic profiling and discriminant taxa, we identified distinct taxa that can distinguish TT from NT. In particular, at the genus level, Escherichia-Shigella, Fusobacterium, Streptococcus, Peptostreptococcus, Parvimonas, Klebsiella, and Gemella were found to be significantly more abundant in TT. The enrichment of *Fusobacterium* and *Streptococcus* in TT has been consistently reported across numerous studies, highlighting their important role in tumor initiation and progression [20, 21]. Notably, *Parvimonas* exhibited a significant positive correlation with the host gene PARVB, which is highly expressed in CRC tissues [22]. Furthermore, *Escherichia-Shigella*, *Peptostreptococcus*, and *Klebsiella* were found to be enriched specifically in CRC patients compared to healthy volunteers in an investigation focusing on intestinal flora composition [23]. *Gemella* which predominantly resides within the oral cavity and upper gastrointestinal tract, was reported to be associated with oral squamous cell carcinoma [24]. In summary, the above findings suggest subtle differences in microbial diversity between TT and NT. Furthermore, both TT and NT exhibit unique taxonomic profiles, each characterized by a dominant genus.

Recognizing that a single taxonomic group might not fully capture microbial differences between TT and NT, we applied hierarchical clustering based on Bray-Curtis distance to construct four co-abundance groups (CAG 1–4). These constructed CAGs were then used for unsupervised clustering of TT and NT samples, resulting in the classification of all samples into six major categories (sample group 1–6). CAG 2 was notably enriched in TT tissues, while CAG 4 was enriched in NT. Sample group 3 and sample group 5 contained predominantly NT, whereas sample group 4 and sample group 6 contained predominantly TT. The CAGs level analyses revealed that sample group 4 and sample group 6 exhibited a higher abundance of CAG 2, sample group 3 had an increased abundance of CAG 3, and sample group 5 was enriched with CAG 4. Further examination revealed that the abundance of CAG 1 in sample group 1 was exceptionally high, nearly 100%, resulting in a low abundance of the remaining CAGs. To address this question, we excluded sample group 1 and conducted the same analysis with the remaining samples. In the remaining samples, we found that CAG 3 was significantly increased in NT. Additionally, CAG 3 and CAG 4 exhibited a positive correlation, though not statistically significant, and both were negatively correlated with CAG 2. Previous studies have demonstrated the feasibility of classifying experimental subjects using bacterial abundance or CAGs. For instance, in a study on colorectal cancer and adjacent normal tissues, K-means clustering was employed to divide the samples into three distinct subgroups [12]. Another similar study clustered the Operational Taxonomic Unit (OTU) hierarchy into six CAGs, subsequently categorizing the samples into multiple distinct subgroups, a process replicated in two additional cohorts [10]. In our study, CAG 1 comprises a substantial number of nonpathogenic or opportunistic pathogens that were widely found in nature or the human body, including *Bacteroides*, *Delftia*, *Enterococcus*, *Klebsiella*, *Proteus*, *and Pseudomonas* [25–30]. CAG 2 includes *Fusobacterium*, *Streptococcus*, *Peptostreptococcus*, and *Parvimonas*, which were reported to promote the occurrence and progression of CRC in various studies. Interestingly, these four genera were assigned to the same CAG which was considered a pathogenic bacterial cluster in the study by Flemer et al. [10]. Campylobacter in CAG 2 was reported to be associated with colorectal and esophageal cancer [31]. In CAG 3 and CAG 4, we identified more bacteria that are considered to be probiotic or nonpathogenic such as *Akkermansia*, *Alistipes*, *Bifidobacterium*, *Blautia*, *Collinsella*, *Faecalibacterium*, *Parabacteroides*, *Prevotella*, *Bacillus*, and *Lactobacillus* [32–41]. Hence, human diseases can be attributed not only to a single pathogen but also to overall changes in the microbiota [42]. For instance, a study on breast cancer described the combination of estrogen in the liver, excretion into the gastrointestinal cavity, conjugation by bacterial β-glucuronidase, reabsorption as free estrogens through the enterohepatic circulation, and distribution to different organs like the breast. These metabolites, produced by several bacteria from the Clostridia and Ruminococcaceae families through estrogen metabolism, may collectively have breast cancer-causing potential [43]. These findings offer insights into flora changes during the transformation from NT to TT in a higher dimension, exploring bacterial interaction from the bacterial clusters, and providing clues to the mechanism of the multi-bacterial joint promotion of CRC occurrence and development.

Based on the previously constructed CAGs, we further investigated the association of CAGs in TT with clinical characteristics. CAG 2 was found to be associated with the microsatellite status of tumors, exhibiting higher abundance in TT associated with microsatellite instability. Previous studies conducted in Japan and the United States have demonstrated a significant correlation between *F. nucleatum* and microsatellite instability [44]. Notably, *Fusobacterium* was seen in our CAG 2 *cohort*. Furthermore, CAG 4 exhibited a positive correlation with CA199 levels. CA199 is a typical marker for gastrointestinal tumors and has high sensitivity for pancreatic cancer diagnosis, as well as aiding in rectal cancer, colon cancer, and primary liver cancer detection [45]. In intrahepatic cholangiocarcinoma cases, *Bacillus anthracis* and *P. azotoformans* were observed to be positively associated with CA199 levels [46], and *Bacillus* was notably seen in our CAG 4 cohort. In addition, as shown in Supplementary Fig. 1C, the distribution difference of TNM stage was observed in CAG 4.

In addition to compositional changes in bacterial taxa, we also observed predicted functional alterations across different groups. We found that the following metabolic pathways which include nucleotide metabolism, lipid metabolism, enzyme metabolism, energy metabolism, carbohydrate metabolism, and amino acid metabolism were enriched in the NT group. Similar findings were reported in previous studies [47–49]. Our findings suggest that microbial changes may impact multiple metabolic pathways including amino acid, lipid, and carbohydrate metabolism that could potentially underlie the transition from NT to TT.

Our research boasts a relatively substantial sample size, contributing to the generation of robust and reliable findings. However, several limitations still need addressing. Firstly, in our cluster analysis, two sample groups could not be accurately classified, possibly due to the heterogeneity of tumor samples in terms of location and subtype. Previous studies have highlighted differences in microbial composition between CRC originating from different locations or subtypes [50, 51]. Secondly, cross-sectional studies emphasize the need for prospective trials to fully elucidate the role of microbiota in CRC. Lastly, as we employed 16 S rRNA gene sequencing for microbiota analysis, we were unable to determine species-level composition and actual genetic functions. Further investigations utilizing shotgun metagenomic sequencing are warranted to unravel the mechanisms underlying CAGs and CRC.

In summary, our research will deepen our understanding of the interactions among multiple bacteria and offer insights into the potential mechanism of NT to TT transition.

### Electronic supplementary material

Below is the link to the electronic supplementary material.

Supplementary Material 1

## Data Availability

The datasets presented in this study can be found in online repositories. The names of the repository/repositories and accession number(s) can be found below: https://www.ncbi.nlm.nih.gov/, PRJNA1068013.
