# Supplementary material for: Tumour-associated and non-tumour-associated bacteria co-abundance groups in colorectal cancer
Source: BMC Microbiol. 2024 Jul 3;24:242. doi: 10.1186/s12866-024-03402-5 (PMC11223424; doi:10.1186/s12866-024-03402-5)
Supplement: Supplementary file 1 — Supplementary Material 1 [file 12866_2024_3402_MOESM1_ESM.pdf]

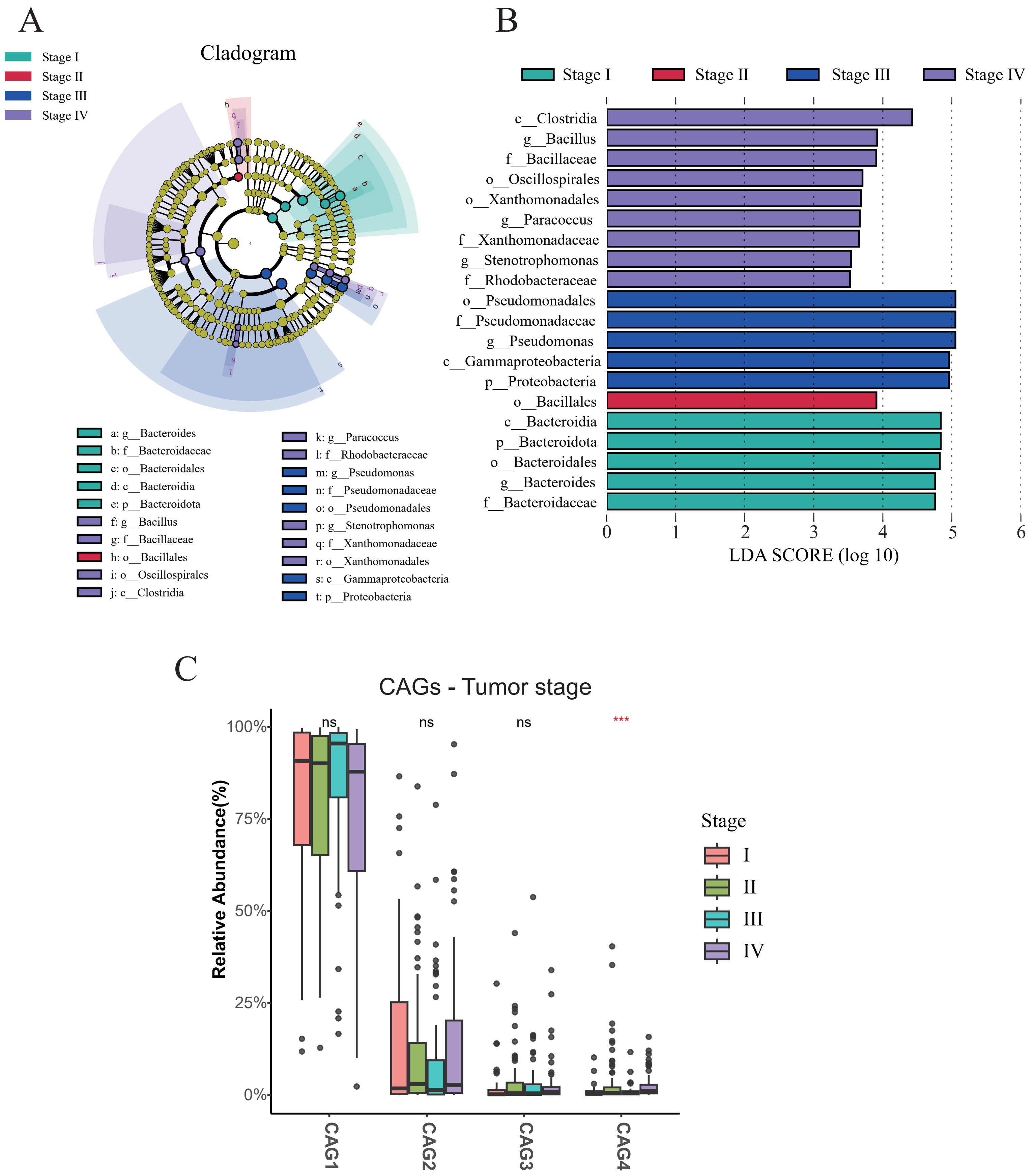

Supplementary Figure 1. Characteristics of the microbiota in different TNM stages from tumor tissue. Cladogram (A) and Linear discriminant analysis effect size (LEfSe) analysis (B) showed the most abundant taxa from the phylum to the genus level among the stage I, stage II, stage III, and stage IV, LDA score threshold >3.5. (C) Boxplots of correlation between CAGs and tumor stage. ****p < 0.0001, ***p < 0.001, **p < 0.01, *p < 0.05.

Supplementary Table 1. Differences in relative abundance of bacterial genera based on Paired Wilcoxon Signed Rank Test or Paired T-test.

Genu	Normal tissue mean(%)	Tumor tissue mean(%)	Paired T-test p value	Paired Wilcoxon p value
Delftia	19.45	15.74	0.002	0.000
Escherichia-Shigella	7.16	13.19	0.000	0.000
Pseudomonas	12.31	10.65	0.027	0.002
Bacteroides	6.81	7.94	0.228	0.639
Fusobacterium	1.03	3.77	0.000	0.000
Streptococcus	1.05	2.16	0.015	0.000
Enterococcus	1.35	1.96	0.146	0.089
Peptostreptococcus	0.81	1.53	0.001	0.000
Parvimonas	0.58	1.23	0.000	0.000
Gemella	0.52	1.08	0.001	0.000
Bifidobacterium	1.54	0.79	0.014	0.206
Klebsiella	0.19	0.73	0.002	0.000
Bacillus	1.12	0.59	0.017	0.103
Campylobacter	0.24	0.57	0.158	0.000
Achromobacter	0.67	0.55	0.324	0.006
Acinetobacter	1.05	0.52	0.003	0.000
Granulicatella	0.19	0.50	0.017	0.000
Blautia	0.80	0.45	0.000	0.000
Parabacteroides	0.68	0.41	0.014	0.000
Porphyromonas	0.19	0.40	0.110	0.000
Prevotella	0.33	0.38	0.516	0.298
Chloroplast	0.52	0.37	0.707	0.260
Faecalibacterium	0.41	0.37	0.525	0.352
Leptotrichia	0.05	0.30	0.061	0.001
Eikenella	0.04	0.28	0.047	0.000
Proteus	0.01	0.25	0.316	0.148
Dialister	0.09	0.18	0.027	0.004
Alistipes	0.39	0.15	0.007	0.000
Collinsella	0.27	0.15	0.023	0.057
Lactobacillus	0.38	0.14	0.015	0.000
Solobacterium	0.06	0.13	0.003	0.000
Ralstonia	0.19	0.12	0.017	0.033
Chryseobacterium	0.19	0.11	0.427	0.000
Anaerococcus	0.10	0.11	0.843	0.042
Hungatella	0.04	0.10	0.012	0.022
Subdoligranulum	0.21	0.09	0.000	0.001

Erysipelatoclostridium	0.19	0.09	0.007	0.033
Holdemanella	0.17	0.09	0.041	0.010
Aquabacterium	0.16	0.08	0.035	0.333
Treponema	0.00	0.08	0.145	0.009
Peptoniphilus	0.01	0.08	0.103	0.142
Veillonella	0.08	0.07	0.770	0.421
Pelomonas	0.12	0.07	0.029	0.033
Lachnospiraceae_UCG-010	0.03	0.06	0.001	0.000
Akkermansia	0.21	0.06	0.121	0.031
Erysipelotrichaceae_UCG-003	0.13	0.06	0.004	0.001
Methylobacterium-Methylorubrum	0.07	0.06	0.534	0.075
Alloprevotella	0.02	0.05	0.228	0.022
Burkholderia-Caballeronia-Paraburkholderia	0.09	0.05	0.013	0.001
Staphylococcus	0.11	0.05	0.094	0.298
Desulfovibrio	0.06	0.05	0.497	0.948
Comamonas	0.06	0.05	0.091	0.000
Stenotrophomonas	0.07	0.05	0.163	0.015
Aggregatibacter	0.01	0.04	0.117	0.000
Eggerthella	0.07	0.04	0.007	0.045
Olsenella	0.01	0.04	0.072	0.002
Bilophila	0.04	0.04	0.838	0.926
Enhydrobacter	0.04	0.03	0.920	0.427
Shewanella	0.00	0.03	0.008	0.000
Phascolarctobacterium	0.04	0.03	0.664	0.193
Filifactor	0.01	0.03	0.069	0.003
Odoribacter	0.05	0.03	0.101	0.019
Morganella	0.00	0.03	0.044	0.002
CAG-352	0.11	0.03	0.053	0.037
UCG-002	0.03	0.03	0.646	0.411
Actinomyces	0.01	0.03	0.323	0.217
Kurthia	0.07	0.03	0.024	0.000
Pyramidobacter	0.01	0.03	0.030	0.004
Mogibacterium	0.02	0.03	0.203	0.043
Corynebacterium	0.12	0.03	0.261	0.001
Flavonifractor	0.03	0.03	0.863	0.449
Ruminococcus	0.05	0.03	0.116	0.435
Christensenellaceae_R-7_group	0.03	0.03	0.414	0.467
Eubacterium_coprostanoligenes_group	0.02	0.02	0.931	0.744

Oscillibacter	0.00	0.02	0.022	0.001
Incertae_Sedis	0.02	0.02	0.497	0.496
Johnsonella	0.00	0.02	0.010	0.003
Paracoccus	0.03	0.02	0.173	0.004
Butyricimonas	0.03	0.02	0.006	0.006
Clostridium_sensu_stricto_1	0.03	0.02	0.124	0.011
UCG-005	0.02	0.02	0.874	0.969
Sutterella	0.03	0.02	0.322	0.751
Turicibacter	0.04	0.02	0.057	0.010
Herbaspirillum	0.02	0.02	0.486	0.004
Haemophilus	0.03	0.02	0.334	0.973
Megasphaera	0.01	0.02	0.197	0.332
UBA1819	0.02	0.02	0.073	0.151
Parasutterella	0.02	0.02	0.734	0.827
Gordonia	0.04	0.01	0.002	0.000
Eubacterium	0.03	0.01	0.117	0.008
Megamonas	0.01	0.01	0.460	0.453
Family_XIII_AD3011_group	0.01	0.01	0.370	0.243
Clostridium_innocuum_group	0.02	0.01	0.031	0.019
Family_XIII_UCG-001	0.00	0.01	0.039	0.001
Thermobifida	0.01	0.01	0.608	0.178
Mitochondria	0.01	0.01	0.851	1.000
Bradyrhizobium	0.02	0.01	0.016	0.016
Trichococcus	0.03	0.01	0.198	0.001
Butyricicoccus	0.02	0.01	0.003	0.016
Hydrogenophilus	0.02	0.01	0.064	0.110
Howardella	0.00	0.01	0.018	0.001
Negativibacillus	0.02	0.01	0.093	0.120
Selenomonas	0.00	0.01	0.080	0.033
Aerococcus	0.06	0.01	0.141	0.000
Jeotgalibaca	0.04	0.01	0.253	0.009
Aeromonas	0.00	0.01	0.158	0.082
Eubacterium_siraeum_group	0.00	0.01	0.209	0.426
Fretibacterium	0.00	0.01	0.089	0.052
Monoglobus	0.01	0.01	0.302	0.059
Flavobacterium	0.01	0.01	0.017	0.005
Lachnospira	0.00	0.01	0.269	0.369
Faecalibaculum	0.14	0.01	0.313	0.348

UCG-003	0.01	0.01	0.464	0.829
Barnesiella	0.01	0.00	0.512	0.385
Lachnospiraceae_ND3007_group	0.01	0.00	0.016	0.003
Sellimonas	0.01	0.00	0.151	0.061
Pandoraea	0.00	0.00	0.861	0.326
Exiguobacterium	0.00	0.00	0.812	0.821
SBR1031	0.01	0.00	0.245	0.126
Xanthomonas	0.01	0.00	0.115	0.134
Castellaniella	0.01	0.00	0.000	0.000
Sphingobium	0.00	0.00	0.849	0.354
Ileibacterium	0.00	0.00	0.689	0.966
Bdellovibrio	0.01	0.00	0.213	0.290
Solibacillus	0.01	0.00	0.134	0.049
Atopostipes	0.00	0.00	0.263	0.363
Lactococcus	0.01	0.00	0.049	0.021
env.OPS_17	0.01	0.00	0.235	0.396
Cloacibacterium	0.00	0.00	0.595	0.333
Sphingobacterium	0.00	0.00	0.194	0.188
Diaphorobacter	0.01	0.00	0.006	0.005
Catenibacterium	0.01	0.00	0.092	0.057
Geobacillus	0.01	0.00	0.195	0.166
Anaerotruncus	0.01	0.00	0.031	0.013
Thauera	0.00	0.00	0.160	0.118
Dechloromonas	0.00	0.00	0.007	0.016
Pajaroellobacter	0.00	0.00	0.821	0.434
Weissella	0.00	0.00	0.091	0.025
Holdemania	0.00	0.00	0.626	0.294
Brevundimonas	0.00	0.00	0.118	0.035
Acidibacter	0.00	0.00	0.124	0.047
Petrimonas	0.00	0.00	0.034	0.019
Caproiciproducens	0.00	0.00	0.149	0.235
PHOS-HE36	0.00	0.00	0.028	0.034

Note: The average relative abundance of 0.00 means the value is less than 0.01%. P value less than 0.05 are marked in red
